# Quality of life after extraction of mandibular wisdom teeth: A systematic review

**DOI:** 10.1016/j.amsu.2022.104387

**Published:** 2022-08-21

**Authors:** Lamiae Hallab, Asma Azzouzi, Bassima Chami

**Affiliations:** Mohammed V University in Rabat Morocco, Morocco

**Keywords:** Quality of life, Mandibular wisdom tooth, Extraction, Systematic review

## Abstract

**Objective:**

The objective of this systematic review was to evaluate the impact of mandibular wisdom tooth extraction on a patient's quality of life “QoL”.

**Methods:**

An electronic search was conducted through September 2021 on MEDLINE database, ELSEVIER- ScienceDirect, Ebsco, Scopus and Google Scholar to collect sufficient articles relevant to our subject. Data were extracted and analyzed from selected studies including study type, sample size and characteristics, duration of the observation after removal wisdom teeth, the questionnaire used for evaluation of this QoL and, the result.

**Results:**

Of 107 studies, fourteen representing 4990 cases met the inclusion criteria. The quality of life has deteriorated but different factors contributed to his improvement. Thus, different instruments have been used in these studies: 24 the OHIP-14, 10 the OHQoLUK, 8 the HRQOL, 2 the EQ-5D-3L QOL, and 1 used UW-QOL.

**Conclusion:**

The extraction of mandibular wisdom teeth has a negative effect on the quality of life during the first postoperative days but improved progressively by following the medical instructions given by the dental surgeon.

## Highlights

• Extraction of mandibular wisdom teeth has a negative effect on the quality of life “QOL” during the immediate postoperative period.
• Several instruments have been used for the assessment of this “QOL”.
• General and local factors were also involved in the improvement or deterioration of this quality of life.

## 1 Introduction

The extraction of mandibular wisdom teeth represents the most frequent surgical procedure performed in oral surgery with a percentage of 5 million per year in the United States [[1], [2], [3], [4],^8^,^14^,^16^]. Different complications are frequently encountered in the majority of the population in the first few days following this extraction such as: osteitis, alveolitis, pain, trismus, edema as well as a difficulty of swallowing [2,3,10,16]. Thus, it should be noted that these complications might significantly lead to deterioration in the quality of life (QoL) during the immediate postoperative period [1,8,9] (Table 5, Table 6).

Quality of life can be defined as “a state of well-being” which is based on two components. The first is the ability to perform daily activities that reflect physical, psychological, and social well-being and the second is the patient's satisfaction with the level of functioning, control of disease, and treatment-related symptoms [15,16].

For the assessment of this quality of life, several instruments have been used. We can identify in the study of Shugars et al. [3] the HRQOL, which allows us to appreciate the perception after the surgical extraction of mandibular wisdom tooth according to 4 domains “oral function, general activity, signs and symptoms, pain”. In addition, Matijevic et al. [7] and Braimah et al. [11] used OHIP-14 or OHQoL-UK [11] to evaluate the quality of life with positive and negative aspects after this surgery.

This systematic review of the literature aimed to determine the impact of the surgical removal of the third molar on physical, psychological, and social well-being by using different instruments. In addition, to expose the different measures, which contribute to his improvement.

## 2 Materials and methods

We conducted this review according to the Cochrane Handbook of Systematic Reviews and Interventions, the PRISMA (Preferred Reporting Items for Systematic Reviews and Meta-Analysis) guidelines, and AMSTAR (Assessing the methodological quality of systematic reviews) guidelines [12,13]. It was registered on PROSPERO (ID: CRD42022319556).

### 2.1 Criteria for considering studies for this review

**Types of studies:** prospective and retrospective studies, observational and randomized clinical trials.

**Types of participants:** Patients in good health who underwent surgical extraction of mandibular wisdom teeth.

**Types of interventions:** Extraction of the mandibular wisdom tooth in different positions: “horizontal, vertical and mesio or disto-position”.

**Types of outcome measures:** The main objective was to determine the severity of quality of life impairment after mandibular wisdom teeth extraction by using different types of questionnaires.

**The primary outcome:** depending on the postoperative days, this QoL differs with a significant deterioration in the 1st days but gradually improves.

**The secondary result:** Several procedures have been reported in the literature to improve the quality of life of patients after mandibular wisdom teeth extraction.

### 2.2 Search methods for identification of studies

#### 2.2.1 Selection of studies

To identify studies included in or considered for this review, we developed detailed search strategies for each database searched until September 2021. Based on the search strategy developed for MEDLINE but revised appropriately for each database. A PICO approach was used in the databases search with MeSH and text words.

The electronic data resources used were “National Library of Medicine, Washington” (MEDLINE-PubMed); the Cochrane Central Register of Controlled Trials (CENTRAL); (CINAHL-EBSCOhost); (ELSEVIER- ScienceDirect), (SCOPUS). The search was limited to human clinical studies and the last electronic search was performed in September 2021. The reference lists of the articles identified were cross-checked for other relevant articles (Table 1).

**Table 1:** Systematic search strategy for study selection.

***Systematic search strategy***	
***Focus question***	What is the effect of surgical removal of mandibular third molar on quality on life in the postoperative days
***Search strategy***	
Population	Patients who underwent surgical extraction of mandibular wisdom teeth
Intervention	#1 (Third mandibular molar extraction) OR (Third mandibular molar removal) OR (Wisdom Tooth removal) OR (Wisdom Tooth extraction)
Comparison	#2 Assessment Quality Of Life
Outcome	Surgical removal of wisdom teeth has a negative impact on the physical, psychological and social well-being of the patients which is evaluated by a questionnaire
***Search combinations***	(#1 AND #2)
***Electronic Database***	MEDLINE and ScienceDirect, Cochrane, Ebsco, Scopus and Google Scholar

#### 2.2.2 Data collection and analysis

Two review authors (LH and BC) separately examined the title and abstract of each article identified by the different search strategies. The authors classified relevant studies.

#### 2.2.3 Inclusion and exclusion criteria

Publications written in English and French were included. While those in Arabic language systemic reviews, studies that did not include questionnaires, and those focusing on upper wisdom teeth were excluded.

#### 2.2.4 Data extraction and management

All studies responding to the inclusion criteria underwent data extraction performed by at least two review authors. Both reviewers used a standardized data extraction sheet with the following parameters: study type, questionnaire quality of life, treatment in the control or placebo group, the total number of patients, and the total duration of observation.

We present the characteristics of trial participants, interventions, and outcomes for the trials in the Characteristics of included studies.

## 3 Results

### 3.1 Study selection

A total of 107 studies were identified. Of this, 13 duplicate articles were excluded, which resulted in 94 articles for analysis. After selected titles and abstracts according to the eligibility criteria required for our study, 74 full-text articles remained, of which 20 were excluded at this stage. Finally, 40 articles comprising 4990 patients were selected for inclusion in our work (Table 2).

**Table 2:**
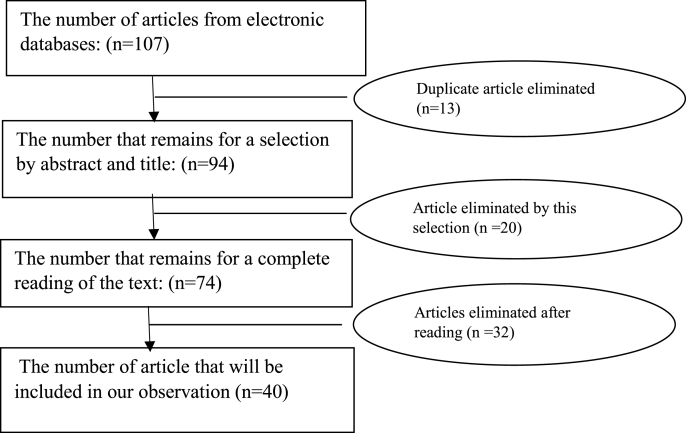
Flow diagram showing the process of inclusion of the studies.

### 3.2 Study results

For the evaluation of the quality of life after removal of mandibular wisdom teeth, different instrumentation has been used. However, only three studies have compared the efficacy of each instrument with the other [3,29,49].

Concerning the different prescriptions, five studies were interested in the prescription of corticosteroids alone [18,22,30] or associated with NSAIDs [5,6], and three included the effect of antibiotic therapy or prophylaxis [26,28,47].

Others covered the results of PRF/PRP [4], kinesiotaping [17,20], hiloterm [24], ice bladder [32], the iodine pad [19](23), surgical site irrigation [27], removal of sutures [33], neurofeedback [34], laser [35](38), bromelain [36], ozone therapy [37] in improving quality of life.

Regarding the general and local factors, seven studies have evaluated the effect of age and sex variation [42](45) [46], smoking, poor oral hygiene [43], the position of the symptomatic or asymptomatic wisdom tooth [21](40) and pericoronitis [41] on the quality of life.

Concerning the postoperative duration and the effect of postoperative instructions on the quality of life eight studies have reported this [1](7) [25](31) [2](39) [44](48).

Features of every single study are reported in Table 3

**Table 3:** Characteristics of the included studies.

Authors	Years	Types of studies	Evaluation criteria	The population	Duration of the observation	Questionnaires	The results
**Osagie O et al** [4]	2021	Prospective randomized study	Clinics	50 patients	1, 3, and 7 days	OHQoL-UK	Postoperative application of PRF “platelet-rich fibrin” at the extraction site of the impacted lower wisdom tooth has a positive impact on oral health related quality of life. In relation to the effect of PRP « platelet-rich plasma” according to this study there was no significant difference with PRF.
				aged between 18 and 55 years			
**Xie L et al** [5]	2021	Randomized, Double-Blind, Placebo-Controlled Clinical Trial	Clinics	60 patients	1–7 days	UK-OHRQoL	Preemptive oral etoricoxib
				aged between 18 and 48 years			(60 mg 30 min before intervention) represent an effective therapeutic approach to improving quality of life following surgical extraction of a lower third molar.
**Braimah RO et al** [6]	2021	Prospective study	Clinics	78 patients	1, 3, 5, 7 and 14 days	UK-OHRQoL	Quality of life was better in the group of patients who received IM co-administration of 8 mg Dexamethasone and 75 mg Diclofenac.Compared to those who just put ice packs extra-orally *trans*-alveolar after extraction of impacted mandibular third molars.
				aged between 20 and 49 years			
**Jaron A et al** [17]	2021	Prospective study	Clinics	100 patients	1–7 days	UW-QoL v4	Kinesio Taping has a considerable impact on the quality of life after the extraction of an impacted third molar.
				aged between 18 and 59 years			
**Larsen MK et al** [18]	2021	Double-blind, split-mouth, randomised controlled trial	Clinics	52 patients	1, 3, 7 days and 1 months	OHIP-14	No significant difference of methylprednisolone or placebo in postoperative sequelae and quality of life after third molar mandibular removal
				aged between 18 and 39 years			
**Lindeboom JA et al** [19]	2021	Prospective randomized controlled trial	Clinics	87 patients	1–7 days	OHIP-14	The insertion of an iodine pad into the postoperative socket decreased pain and impact on oral health-related quality of life in the first postoperative week.
				Average age 26.47 years			
**Doni B R et al** [21]	2021	Descriptive cross-sectional study	Clinics	246 patients	3 months	OHIP-14	Quality of life after removal of mandibular third molar in asymptomatic patient was better compared to those who were symptomatic.
				Aged between 15 and 58 years			
**Erdil A et al** [20]	2020	Randomized, controlled clinical trial	Clinics	82 patients	2, 7 days	OHIP-14	The combination of Kinesio taping with injection of corticosteroides in preoperatively or prescription of anti-inflammatory in postoperative provide results in terms of trismus,
				Aged between 18 and 65 years			edema, and QoL after third molar extraction.
**Ai Lyn Lau A et al** [22]	2020	Randomized, controlled, double-blinded trial	Clinics	130 patients	2, 7 days	OHIP-14	Submucosal administration of dexamethasone has a positive impact on oral health related quality of life and postoperative swelling, pain and trismus after third
				Aged between 16 and 40 years		OHRQoL	molar surgery.
**Tuk GJ et al** [23]	2019	Prospective, crossover, randomized controlled study	Clinics	54 patients	1–7 days	OHRQoL	The administration of an iodine-containing tampon in the socket after the extraction of impacted mandibular third molars has a positive impact on the oral health related quality of life.
				Average age 25.1 years			
**Beech AN et al** [24]	2018	Observational study	Clinics	30 patients	1–7 days	EQ-5D-3L QOL	The use of a home facial cooling system “The Hilotherm” provides an improvement in the quality of life after extraction of the impacted mandibular wisdom tooth.
				Aged between 18 and 25 years			
**Ibikunle AA et al** [25]	2017	Observational study	Clinics	124 patients aged between 18 and 51 years	1–7 days	OHIP-14	The patients' quality of life was impaired on days 1 and 3 after extraction of the impacted mandibular wisdom tooth, but was significantly improved on day 7 postoperatively.
**Essen A et al** [26]	2017	Retrospective study based on a graph	Clinics	62 patients aged between 18 and 40 years	1–5 days	OHIP-14	The preoperative prescription of the antibiotic combination Amoxicillin/Clavulanic acid would have the same effect on the quality of life when using amoxicillin alone.
**Fennis JP et al** [27]	2017	Randomized controlled trial	Clinics	280 patients	1–7 days	OHIP-14	Irrigation of the surgical site with tap water using a curved syringe after extraction of the impacted mandibular wisdom tooth is effective in reducing the risk of inflammatory complications.
				aged under than 26 years old			
**Braimah RO et al** [28]	2017	Observational study	Clinics	135 patients	1–7 days	UK-OHRQoL	A pre- and postoperative prescription of amoxicillin 875 mg combined with clavulanic acid 625 mg provides an improvement in quality of life after extraction of the impacted mandibular wisdom tooth. This is in contrast to antibiotic prophylaxis with amoxicillin 875 mg and clavulanic acid 125 mg.
				aged between 18 et 35 years			
**Beech AN et al** [29]	2017	Observational study	Clinics	40 patients	1–7 days	EQ5D3L	The generic EQ3D3L instrument appears to be less used because it does not include the objective measures of pain and swelling contrary to OHIP-14.
				aged between 18 and 61 years		OHIP-14	
**Ibikunle AA et al** [30]	2016	Prospective study	Clinics	168 patients	1–7 days	OHIP-14	Intravenous injection of Prednisolone preoperatively improves quality of life after extraction of the impacted mandibular wisdom tooth compared to oral taken of the same drug.
				aged between 21 and 31ans			
**Braimah RO et al** [31]	2016	Prospective study	Clinics	135 patients aged between 18 and 25ans	1–7 days	UK-OHRQoL	There is a deterioration of the quality of life especially during the first postoperative days.
**Ibikunle AA et al** [32]	2016	Prospective randomized clinical trial	Clinics	139 patients	1–7 days	OHIP-14	Patients who used the Ice Pack at the operative site expressed a better quality of life after extraction of the impacted mandibular wisdom tooth than those who did not.
				Aged between 18 and 49ans			
**Rodanant P et al** [33]	2016	Prospective randomized controlled trial	Clinics	30 patients aged between 17 and 30ans	1–7 days	OHIP-14	The quality of life after removal of the suture from the surgical site after the 3rd or 7th postoperative day was the same, under the condition of avoiding any risk of contamination by unsatisfactory oral hygiene.
**Aravena P et al** [2]	2016	Prospective study	Clinics	106 patients Older than 15 years	1–7 days	HRQOL-sp	The quality of life after extraction of the impacted mandibular wisdom tooth was interfered especially in the first days after the operation. But several factors contributed to a good improvement:
							Postoperative prescriptions, rest, etc.
**Chisci G et al** [34]	2015	Prospective study	Clinics	10 patients	1–14 days	HRQOL	The technique (Neuronal feedback(NF)) allows to minimize the injury of the inferior alveolar nerve in case of contact of the impacted lower wisdom tooth. It has also been shown that a long time of surgery leads to postoperative complications and an altered quality of life.
**Matijevic M et al** [7]	2014	Observational study	Clinics	108 patients	1–30 days	OHIP-14	Postoperative oral instructions can significantly improve the quality of life after extraction of the impacted mandibular wisdom tooth.
				average age of 32 years			
**Batinjan G et al** [35]	2014	Prospective study	Clinics	40 patients	1–7 days	OHIP-14-CRO	Laser (antimicrobial photodynamic treatment “aPDT”) allows better healing of the operative wound, a diminution of pain, swelling, and temperature especially in the 5th day after the extraction of the impacted mandibular wisdom tooth.
				Aged between 19 and 32ans			
**Majed OW et al** [36]	2014	Prospective study	Clinics	45 patients	1–7 days	OHIP-14	Bromelain 250 mg taken pre and postoperatively for 4 days showed a significant improvement in quality of life compared to diclofenac sodium.
				Aged between 18 and 35ans			
**Kazancioglu HO et al** [37]	2013	Prospective study	Clinics	60 patients	1–7 days	OHIP-14	Ozone therapy showed a significant improvement in quality of life and a reduction in pain after extraction of the impacted mandibular wisdom tooth. Moreover, this treatment had no effect on postoperative swelling and trismus.
				Aged between 18 and 25ans			
**Sierra SO et al** [38]	2013	Randomized clinical trial	Clinics	60 patients	1–7 days	OHIP-14	Intra- and extra-oral low power laser (LLLT) allows good healing, a significant reduction of pain, trismus, and swelling and improved quality of life on days 2 and 7 after extraction of the impacted mandibular wisdom tooth.
				Aged between 18 and 30ans			
**Sancho-Puchades M et al** [39]	2012	Prospective study	Clinics	50 patients	1–7 days	HRQOL-sp	The extraction of the impacted mandibular wisdom tooth affects the quality of life especially in the first 5 days. Intraoperative conscious sedation with Midazolam provides comfort for the patient but has no effect in the postoperative period.
				Aged between 18 and 25ans			
**Negreiros RM et al** [40]	2012	Prospective study	Clinics	86 patients	1–7 days	OHIP-14	A compromised position of the impacted mandibular wisdom tooth (e.g. disto-angular) involves a complex technique for this extraction, which results in a negative alteration of the postoperative quality of life.
				aged between 18 and 25 years			
**Shenan B et al** [41]	2012	Prospective study	Clinics	60 patients	3 months	OHIP-14	Quality of life is negatively affected in patients with minor pericoronitis symptomatology after extraction of impacted mandibular wisdom teeth.
				aged between 18 and 35 years			
**Ceib P et al** [42]	2010	Prospective study	Clinics	958 patients aged between 14 and 40 years	1–14 days	HRQOL	Patients younger than 21 years of age recover more quickly and therefore have a better quality of life compared to those who are older.
**Larrazabal C et al** [43]	2010	Prospective study	Clinics	50 patients	1–7 days	OHIP-14	Patients who did not brush their teeth and who smoked cigarettes pre- and postoperatively had intolerable pain in the first 24 h after extraction of the impacted mandibular wisdom tooth.
				Aged between 18 and 39 years			
**Sato RF et al** [44]	2009	Prospective study	Clinics	128 patients	1–7 days	HRQOL	The quality of life of patients after extraction of impacted mandibular wisdom teeth was deficient in the first 3 days postoperatively and which tended to improve with time.
				Aged between 16 and 40 years			
**Deepti C et al** [1]	2009	Randomized controlled trial	Clinics	72 patients aged between 18 and 45 years	1–7 days	OHIP-14	There was a significant deterioration in quality of life during the first 5 days after extraction of the impacted mandibular wisdom tooth, which improved after the 6th day. The use of these two questionnaires in this study identified that there is no difference between them.
						OHQoLUK-16	
**Chuang SKEt al** [45]	2007	Prospective cohort study	Clinics	4004 patients	1–7 days	OHIP-14	There is an increased risk of complications and deterioration of quality of life in patients over 25 years of age compared to those who were younger.
				aged between 13 et 89 ans			
**Shugars DA et al** [3]	2006	Prospective observational study	Clinics	63 patients	1–7 days	OHIP-14	The use of these two instruments showed significant results in determining quality of life after extraction of the impacted mandibular wisdom tooth.
				under than 25 years old		OHQoL-UK	
**Colorado-Bonnin M et al** [46]	2006	Objective observational study	Clinics	105 patients average age 25.1 ans	1–7 days	HRQOL-sp	Women experienced more pain than men, especially in the first 3 days after extraction of the impacted mandibular wisdom tooth. In addition, patients who were followed by telephone and were able to follow the instructions had an improvement in their quality of life postoperatively.
**Stavropoulos MF et al** [47]	2006	Prospective study	Clinics	63 patients	1–14 days	HRQOL	Topical application of Minocycline or Ampicillin improves the quality of life after extraction of the impacted mandibular wisdom tooth.
				Aged between 18 and 25 years			
**White RP et al** [48]	2003	Observational study	Clinics	740 patients	1–14 days	HRQOL	After extraction of the impacted mandibular wisdom tooth, most patients reported pain, swelling and deterioration of their quality of life. But this tended to decrease until it disappeared over time.
				Aged between 14 and 40 years			
**Colman MC et al** [49]	2003	Prospective observational study	Clinics	100 patients	1–7 days	OHIP-14	The OHIP-14 instrument was more reliable and significant in measuring quality of life after extraction of the impacted mandibular wisdom tooth. This was explained by the significant difference in scores and much more severe changes in the level of perception.
				under than 26 years old		OHQOL-UK	

### 3.3 Risk of bias (quality) assessment (Table 4, Table 5)

**Table 4:** Appraisal tool for Cross-Sectional Studies (AXIS) [50].

	Doni B R et al. [21]		
	YES	NOT	Do not know/comment
Introduction			
1 Were the aims/objectives of the study clear?	+		
Methods			
2 Was the study design appropriate for the stated aim(s)?	+		
3 Was the sample size justified?	+		
4 Was the target/reference population clearly defined? (Is it clear who the research was about?)	+		
5 Was the sample frame taken from an appropriate population base so that it closely represented the target/reference population under investigation?	+		
6 Was the selection process likely to select subjects/participants that were representative of the target/reference population under investigation?	+		
7 Were measures undertaken to address and categorise non-responders?	+		+
8 Were the risk factor and outcome variables measured appropriate to the aims of the study?	+		
9 Were the risk factor and outcome variables measured correctly using instruments/measurements that had been trialled, piloted or published previously?	+		+
10 Is it clear what was used to determined statistical significance and/or precision estimates? (eg, p values, CIs)			
11 Were the methods (including statistical methods) sufficiently described to enable them to be repeated?			
Results			
12 Were the basic data adequately described?	+		
13 Does the response rate raise concerns about non-response bias?			+
14 If appropriate, was information about non-responders described?		+	
15 Were the results internally consistent?	+		
16 Were the results for the analyses described in the methods, presented?	+		
Discussion			
17 Were the authors' discussions and conclusions justified by the results?	+		
18 Were the limitations of the study discussed?		+	
Other			
19 Were there any funding sources or conflicts of interest that may affect the authors' interpretation of the results?		+	
20 Was ethical approval or consent of participants attained?	+

**Table 5:** Revised Cochrane risk-of-bias tool for randomized trials (RoB 2) [51].

Study	Risk of Bias Domains					
	D1	D2	D3	D4	D5	Overall
**Xie L et al** [5] **2021**						
	**-**	**+**	**X**	**+**	**+**	**X**
**Larsen MK et al** [18] **2021**						
	**+**	**-**	**-**	**+**	**+**	**-**
**Lindeboom JA et al** [19] **2021**						
	**-**	**-**	**+**	**+**	**+**	**+**
**Erdil A et al** [20] **2020**						
	**+**	**+**	**-**	**+**	**-**	**-**
**Ai Lyn Lau A et al** [22] **2020**						
	**+**	**+**	**-**	**+**	**+**	**+**
**Fennis JP et al** [27] **2017**						
	**+**	**-**	**-**	**-**	**+**	**-**
**Ibikunle AA et al** [32] **2016**						
	**+**	**+**	**-**	**+**	**+**	**+**
**Rodanant P et al** [33] **2016**						
	**-**	**+**	**+**	**+**	**+**	**+**
**Sierra SO et al** [38] **2013**						
	**+**	**+**	**+**	**-**	**+**	**+**
**Deepti C et al** [1] **2009**						
	**-**	**X**	**-**	**-**	**+**	**X**

Domains: Judgement:
D1: Bias arising from the randomization process
**X**
D2: Bias due to deviations from intended intervention High
**-**
D3: Bias due to missing outcome data Some concerns
**+**
D4: Bias in measurement of the outcome Low
D5: Bias in selection of the reported result

**Table 6:** Risk of bias for included studies: NIH Quality Assessment Tool for Observational Cohort studies [52].

NIH Quality Assessment Tool	References of the articles																													
	2	3	4	6	7	17	21	23	24	25	26	28	29	30	31	34	35	36	37	39	40	41	42	43	44	45	46	47	48	49
1 Was the research question or objective in this paper clearly stated?	**Y**	**Y**	**Y**	**Y**	**V**	**Y**	**Y**	**Y**	**Y**	**Y**	**Y**	**Y**	**Y**	**Y**	**Y**	**Y**	**Y**	**Y**	**Y**	**Y**	**Y**	**Y**	**Y**	**Y**	**Y**	**Y**	**Y**	**Y**	**Y**	**Y**
2 Was the study population clearly specified and defined?	**Y**	**Y**	**Y**	**Y**	**V**	**N**	**N**	**Y**	**Y**	**Y**	**Y**	**N**	**Y**	**Y**	**Y**	**N**	**Y**	**Y**	**N**	**N**	**Y**	**Y**	**N**	**Y**	**Y**	**Y**	**Y**	**Y**	**Y**	**Y**
3 Was the participation rate of eligible persons at least 50%?	**Y**	**Y**	**Y**	**Y**	**V**	**N**	**Y**	**Y**	**Y**	**N**	**Y**	**Y**	**Y**	**N**	**Y**	**Y**	**Y**	**Y**	**Y**	**Y**	**Y**	**Y**	**Y**	**Y**	**Y**	**Y**	**Y**	**Y**	**Y**	**Y**
4 Were all the subjects selected or recruited from the same or similar populations (including the same time period)? Were inclusion and exclusion criteria for being in the study prespecified and applied uniformly to all participants?	**Y**	**Y**	**Y**	**Y**	**N**	**Y**	**Y**	**Y**	**Y**	**Y**	**Y**	**Y**	**Y**	**Y**	**Y**	**Y**	**Y**	**Y**	**N**	**Y**	**Y**	**Y**	**Y**	**Y**	**Y**	**Y**	**Y**	**Y**	**Y**	**Y**
5 Was a sample size justification, power description, or variance and effect estimates Provided?	**Y**	**Y**	**Y**	**Y**	**Y**	**Y**	**Y**	**Y**	**Y**	**Y**	**Y**	**Y**	**Y**	**Y**	**Y**	**N**	**Y**	**Y**	**Y**	**Y**	**Y**	**Y**	**N**	**Y**	**N**	**Y**	**Y**	**Y**	**Y**	**Y**
6 For the analyses in this paper, were the exposure(s) of interest measured prior to the outcome(s) being measured?	**N**	**N**	**N**	**Y**	**N**	**N**	**Y**	**Y**	**N**	**N**	**N**	**Y**	**Y**	**Y**	**Y**	**N**	**N**	**N**	**Y**	**N**	**Y**	**Y**	**Y**	**Y**	**Y**	**Y**	**Y**	**N**	**N**	**N**
7 Was the timeframe sufficient so that one could reasonably expect to see an association between exposure and outcome if it existed?	**Y**	**Y**	**Y**	**Y**	**Y**	**Y**	**Y**	**Y**	**Y**	**Y**	**Y**	**Y**	**Y**	**Y**	**Y**	**Y**	**Y**	**Y**	**N**	**N**	**Y**	**Y**	**N**	**Y**	**Y**	**Y**	**Y**	**Y**	**Y**	**Y**
8 For exposures that can vary in amount or level, did the study examine different levels of the exposure as related to the outcome (e.g., categories of exposure, or exposure measured as continuous variable)?	**Y**	**N**	**Y**	**Y**	**Y**	**Y**	**Y**	**Y**	**N**	**Y**	**N**	**Y**	**Y**	**Y**	**Y**	**Y**	**N**	**Y**	**Y**	**Y**	**Y**	**Y**	**N**	**N**	**Y**	**Y**	**Y**	**N**	**Y**	**N**
9 Were the exposure measures (independent variables) clearly defined, valid, reliable, and implemented consistently across all study participants?	**N**	**Y**	**Y**	**Y**	**Y**	**N**	**Y**	**Y**	**Y**	**Y**	**Y**	**Y**	**Y**	**Y**	**Y**	**N**	**Y**	**Y**	**N**	**N**	**Y**	**Y**	**Y**	**Y**	**Y**	**Y**	**Y**	**Y**	**Y**	**Y**
10 Was the exposure(s) assessed more than once over time?	**Y**	**Y**	**N**	**Y**	**Y**	**Y**	**Y**	**Y**	**Y**	**N**	**Y**	**Y**	**Y**	**Y**	**Y**	**N**	**Y**	**N**	**Y**	**Y**	**Y**	**Y**	**Y**	**Y**	**Y**	**Y**	**Y**	**Y**	**N**	**Y**
11 Were the outcome measures (dependent variables) clearly defined, valid, reliable, and implemented consistently across all study participants?	**N**	**Y**	**Y**	**Y**	**N**	**Y**	**Y**	**Y**	**Y**	**Y**	**Y**	**Y**	**Y**	**Y**	**Y**	**Y**	**Y**	**Y**	**N**	**Y**	**Y**	**Y**	**N**	**Y**	**Y**	**Y**	**Y**	**Y**	**Y**	**Y**
12 Were the outcome assessors blinded to the exposure status of participants?	**Y**	**N**	**N**	**N**	**Y**	**Y**	**Y**	**N**	**N**	**N**	**N**	**N**	**N**	**Y**	**N**	**Y**	**N**	**N**	**Y**	**N**	**N**	**N**	**N**	**N**	**N**	**N**	**N**	**N**	**N**	**N**
13 Was loss to follow-up after baseline 20% or less?	**N**	**N**	**N**	**N**	**Y**	**N**	**N**	**N**	**N**	**N**	**N**	**N**	**Y**	**N**	**N**	**Y**	**N**	**N**	**Y**	**Y**	**N**	**N**	**N**	**N**	**N**	**N**	**N**	**N**	**N**	**N**
14 Were key potential confounding variables measured and adjusted statistically for their impact on the relationship between exposure(s) and outcome(s)?	**N**	**Y**	**N**	**N**	**Y**	**Y**	**N**	**N**	**Y**	**N**	**Y**	**N**	**N**	**N**	**N**	**N**	**Y**	**N**	**N**	**Y**	**N**	**Y**	**Y**	**N**	**N**	**N**	**N**	**Y**	**N**	**Y**
**Y: Yes/N: No**																														

## 4 Discussion

The extraction of the impacted mandibular wisdom tooth creates an alteration in the quality of life in the patients postoperatively. This notion of quality of life includes several distinct parameters that describe more precisely the perception of the patient in front of this extraction while taking into account their worries, expectations, and several factors that improve or deteriorate their postoperative period. In relation to the functional limitation: Deepti C et al. [1], Aravena P et al. [2] as well as Shugars DA et al. [3], have represented this after the extraction of the mandibular wisdom teeth by several components. These include difficulty in working, performing sports and leisure activities, discomfort in opening the mouth, which may worsen with the installation of trismus, and difficulties in pronouncing words.

Regarding pain, several authors in particular Xie L et al. [5], Braimah RO et al. [6], Lindeboom JA et al. [19], and Ai Lyn Lau A et al. [22] have discussed the value of preoperative prescription of anti-inflammatory drugs or the use of an iodine tampon in the postoperative socket for pain reduction. We also distinguish the physical disorder represented by a change in diet, the psychological suffering that leads to a temporary depression, but which will decrease until it disappears from the 3rd postoperative day according to most authors [1,3,11].

Now, to assess the impact of mandibular third molar extraction on patient quality of life, the studies in this work have used specific instruments such as OHIP-14, HQoLUK, HRQOL, EQ-5D-3L QOL, and UW-QOL.

Regarding the scoring systems, the higher scores of OHIP-14, and HRQOL was correlated with a negative impact on quality of life, especially from day 1 to day 7 postoperatively.

This finding could be explained by the difficulty of the operation involving osteotomy, separation, and incision as well as possible complications such as trismus, edema, and pain associated with surgical removal of the mandibular third molar [25,31,48].

Currently, when the impact of this extraction on quality of life was analyzed separately for each domain, the domain “physical pain” was mostly recorded by patients (91%) [1,6,22,43].

The present results reveal that pain seems to be the main reason for the deterioration of quality of life after this extraction, mainly on the 1st postoperative day [11,48], and decreasing linearly during the follow-up. These results may provide a source of information for clinical planning when considering prescribing analgesics for faster patient recovery.

Many therapies have been proposed by several authors whose goal is to control postoperative pain and ensure a better quality of life such as: “aPDT laser [35], also the low-powered one (LLLT) [39]", ozone therapy [37] and/or hilotherapy [25]. Medication in the form of “intravenous injection of prednisolone [18] and submucosal dexamethasone [5] or even Bromelain [36] etc.

## 5 Conclusion

In summary, many studies have been conducted on the extraction of impacted mandibular wisdom teeth, and more specifically those evaluating the clinical quality of life after this extraction. Thus, the difference between these studies, notably the sample size, the protocols of realization, the duration of the study, and the criteria of judgment, allows a more precise exploration of this quality of life in all these parameters.

In the present work, a synthetic conclusion can be formulated: the extraction of impacted mandibular wisdom teeth has a negative effect on the quality of life during the first postoperative days but improves progressively by following good postoperative instructions.

## Provenance and peer review

Not commissioned, externally peer-reviewed.

## Compliance with ethical standards

This research involved human participants. This was a retrospective analysis of published cases and did not require informed consent. Ethics approval and consent to participate were not included in this review.

## Ethical approval

Not applicable (Systematic Review).

## Please state any sources of funding for your research

None.

## Author contribution

Dr HALLAB Lamiae designed the concept, analyzed and interpreted the findings, wrote and reviewed the final paper under the supervision of Prof CHAMI Bassima Dr AZZOUZI Asma have also contributed to the realization of this work.

## Please state any conflicts of interest

None.

## Registration of research studies

1. Name of the registry: PROSPERO
2. Unique Identifying number or registration ID: CRD42022319556
3. Hyperlink to your specific registration (must be publicly accessible and will be checked):

## Guarantor

Hallab Lamiae.

## Consent

This research involved human participants. This was a retrospective analysis of published cases and did not require informed consent. Ethics approval and consent to participate were not included in this review.
